# Piloting a one-day parent-only intervention in the treatment of youth with anxiety disorders: child and family-level outcomes

**DOI:** 10.1186/s13034-023-00702-y

**Published:** 2024-01-13

**Authors:** Vanessa E. Cobham, Sarah R. Radtke, Ingrid Hawkins, Michele Jordan, Nasriah Rizman Ali, Thomas H. Ollendick, Matthew R. Sanders

**Affiliations:** 1 School of Psychology, University of Queensland, St. Lucia, QLD 4072 Australia; 2 Virginia Tech: Virginia Polytechnic Institute and State University, Blacksburg, USA

**Keywords:** Parenting, Children, Anxiety disorders, Intensive approaches

## Abstract

**Objective:**

Parent-only cognitive-behavioural therapy (CBT) interventions have promise for youth with anxiety disorders. Fear-Less Triple P (FLTP) is one such intervention that has been found comparable to child-focused CBT. Although traditionally administered in six sessions, a one-day workshop format of FLTP was developed to improve accessibility. The current study compared the effectiveness of the six-session and one-day workshop formats.

**Method:**

Seventy-three youth (mean age, 8.4 years; 74% male) were randomized to traditional FLTP (6-week group) or the one-day workshop format. Anxiety diagnostic status, self- and parent-reported anxiety symptoms scores, independent evaluator-rated improvement, treatment satisfaction, and measures of family functioning were included to assess treatment outcome. Data were collected prior to treatment, and 1-week, 6-months, and 12-months following treatment.

**Results:**

Both conditions resulted in significant improvement in child anxiety symptom scores per parent report (on both questionnaire and diagnostic interview measures). Furthermore, significant decreases in sibling anxiety were observed in both treatment conditions. There were no statistically significant differences between conditions on any outcome measure.

**Conclusions:**

Results of this study add to the growing evidence that brief, low-intensity, parent-only interventions can effectively target child psychopathology. These brief interventions are ideal for families for whom the resources and time required to commit to a standard multi-week intervention are prohibitive.

*Registration of Clinical Trials*: This trial was registered with the Australian and New Zealand Clinical Trials Registry (ACTRN 12615001284550).

## Introduction

Childhood anxiety disorders represent a significant public health challenge and are associated with poor social, academic and health outcomes [1], family dysfunction [2], and significant economic burden [3]. They are the most common mental health problem experienced by children—with a worldwide prevalence rate of 6.5% [4]. Untreated, childhood anxiety disorders have a poor prognosis [5], predict a range of other mental health problems including depressive and substance use disorders in adolescence and adulthood [6], and are associated with poor occupational outcomes [7].

More than three decades of research have demonstrated that psychological interventions are efficacious in the treatment of childhood anxiety [8]. The “gold-standard” intervention, child-focused CBT (typically 10–16 sessions in duration), has consistently been shown to be an effective treatment when compared to waitlist and placebo controls across meta-analyses [9–11]. However, the meta-analyses highlight two concerning facts: (1) a significant proportion of anxious youth do not respond to their initial course of treatment, and (2) in spite of some promising indications of sustained improvements [12], relapse in the long-term appears to be common [13]. The assumption that child-focused CBT does indeed represent the gold-standard intervention has been further challenged by the most recent Cochrane review examining child-focused CBT in the treatment of childhood anxiety [11]. The review concludes that, while CBT is—in the short-term—an effective treatment compared to wait list or no treatment control conditions, there is little to no evidence that CBT is superior to treatment as usual or alternative treatments (though the authors note that the amount and quality of evidence for this latter finding limits the confidence that should be placed in it). The authors also note that, “we still know little about how best to efficiently improve outcomes” ([11], p. 2).

The role of parents in the etiology of childhood anxiety (e.g., [14, 15]) would appear to offer one avenue for efficiently improving outcomes for children with anxiety disorders and has resulted in a body of research evaluating parent + child focused interventions. However, meta-analyses do not support the intuitive hypothesis that these interventions will produce superior outcomes compared to child-focused CBT [10, 11]. Most recently, the role of parents in treatment has been approached differently, with parent-only interventions receiving increased attention. Across a wide range of emotional and behavioural difficulties in children, parent-only interventions have been found to be both valued by and experienced as acceptable by parents [16]. Randomized controlled trials (RCTs) have provided encouraging results for studies evaluating various parent-only interventions in the treatment of childhood anxiety. This includes therapist-supported bibliotherapy [17, 18], as well as clinic-based, parent-only interventions ranging from six [19] to 12 weekly sessions [20]. However, many of these studies are limited by small sample sizes (the studies cited here had sample sizes ranging from 49 to 194) and a lack of long-term follow-up (of the eight studies cited here, three reported only post-treatment follow-up, two reported 6-month follow-up and three reported 12-month follow-up data). That being noted, studies have shown that parent-only interventions produce: (1) superior outcomes compared to a waitlist control [19, 21, 22]; (2) equivalent results to a parent + child intervention [22, 23]; and (3) noninferior results to child-focused CBT [24]. A recent meta-analysis of 29 studies concluded that parent-only interventions have a significant treatment effect when compared to waitlist controls; however, no differences were found when compared to active interventions [25]. Thus, parent-only interventions appear to be a promising way to treat childhood anxiety disorders, offering several potential benefits including the capacity to directly target hypothesised anxiety-maintaining parent behaviours.

Interestingly, regardless of the nature of the intervention, treatment outcomes have tended to focus solely on the “identified” anxious child, as opposed to family-level outcomes. Parental anxiety is the traditional exception to this trend, and some recent studies have examined family functioning as an outcome. One outcome that, to the best of our knowledge, has not been reported upon is sibling anxiety—despite the fact that 12% of siblings of children being treated for an anxiety disorder meet criteria for a previously undiagnosed anxiety disorder themselves [26]. Interest in the effects of treatment on siblings is justified because siblings are among the most important developmental influences on child development. Siblings impact each other in many ways, which include but are not limited to, influencing each other’s acquisition of interpersonal skills [27], emotional development and adjustment [28], development of mental health and behavioural problems [29], and providing protection against the adverse effects of marital discord [30]. Hence, in this study, we examined the effects of the parent focused intervention on the siblings of target children as well.

The current study evaluated two different versions of a parent-only intervention (Fear-Less Triple P; FLTP; [31]): a standard 6-week group format and a one-day intensive workshop format. In a previous study, it has been shown that the 6-week group format produced diagnostic outcomes that were superior to a waitlist control comparison; and that were comparable to outcomes reported in other studies examining child-focused CBT, although it must be noted that FLTP has not yet been compared with either treatment as usual or an active control [19]. At six sessions (~ 9 h clinical contact), FLTP is already briefer than the majority of parent-only interventions (typically 8–12 sessions).

The one-day workshop format (~ 6 h clinical contact) was developed to address the most significant issue facing the field of child anxiety treatment outome research. Namely, efficacious psychological interventions do not have a meaningful public health impact [11]. Community studies suggest that, while the proportion of youth seeking treatment for anxiety has increased [32] in recent years, only a very small proportion are able to access specialist mental health support, with an even smaller proportion (~ 2%) receiving an evidence-based intervention such as child-focused CBT [33]. Common barriers include parents’ perceptions of the social stigma for their children, cost (money and time), and access to care. Recent trends in psychotherapy research have witnessed the development and use of brief and more intensive approaches (brief, intensive and concentrated [BIC] protocols [34]) that are designed to reach more children and their families, to be more efficient, and to be more cost-effective. CBT, given its evidentiary support and its sound underlying therapeutic principles, is uniquely positioned to be in the foreground of this movement. In general, these approaches have modified more traditional or conventional CBT approaches by reducing either the number of sessions or the time period over which the sessions are delivered. Such programs have been developed for youth with specific phobia, obsessive–compulsive disorder, and separation anxiety disorder [34]. However, at this point, a comparable transdiagnostic intervention for anxiety disorders more broadly does not exist. This is especially important in as much as current evidence suggests that clinical contact time is not related to children’s outcomes [11]; and indeed, that the number of treatment sessions/weeks is significantly and negatively related to youth’s post-treatment outcomes [35].

In this study, we evaluated the efficacy of the one-day Fear-Less workshop (a BIC protocol), with the 6-week group program as the control. Primary outcomes related to anxiety diagnostic status. Secondary outcomes included: parent and child ratings of anxiety, independent evaluator (IE)-rated improvement, parent ratings of anxiety-enhancing parenting, parent-rated treatment satisfaction, parent ratings of family functioning, parent anxiety and stress, and anxiety for the sibling closest in age to the identified child. It was hypothesised that:

1. The workshop and the 6-week program would each result in comparable reductions in child anxiety symptoms (as assessed by questionnaire, diagnostic interview and IE ratings of improvement), which would be maintained at follow-up.
2. The workshop and the 6-week program would be highly acceptable to parents. The question of whether they would be equally acceptable to parents was viewed as exploratory in nature.

Finally, in the absence of sufficient research in the area of parent-only interventions—we aimed to explore the impact of the workshop and the 6-week program on sibling anxiety, parental anxiety and stress, and family dysfunction.

## Methods

### Transparency and openness

This research meets Level 1 (Disclosure) for all eight aspects of research planning and reporting of the TOP Guidelines as well as Level 2 (Requirement) for data citation, design and analysis transparency, and study and analysis plan preregistration. We report how we determined our sample size, all data exclusions (if any), all manipulations, and all measures in the study, and we follow Journal Article Reporting Standards (JARS) [36]). All data, analysis code, and research materials are available upon request from the authors. Data were analyzed using IBM Statistics SPSS Version 25 [37]. The trial—which was begun in 2015—was listed with the Australian and New Zealand Clinical Trials Registry (ACTRN 210 12615001284550), and included a basic analysis plan.

### Participants

Participant families were recruited through the media and local schools in metropolitan Brisbane between September 2015 and July 2017. Interestingly, 12 participant families came from suburbs designated as ‘rural and regional’ by the Australian Department of Home Affairs. To be included in the study, children were required to be 7–14 years of age and to meet diagnostic criteria for a primary diagnosis of a DSM-5 [38] anxiety disorder. The exclusion criteria were (a) parent is unable to understand and participate in the treatment; (b) child is concurrently receiving ongoing treatment for anxiety; and (c) child has a significant physical or intellectual impairment. Primary anxiety disorder diagnoses included separation anxiety disorder (*n* = 7, 9.6%), social anxiety disorder (*n* = 19, 26.0%), specific phobia (*n* = 20, 27.4%), and generalized anxiety disorder (*n* = 27, 37.0%); there were no statistical differences in the number of children with each primary anxiety disorder diagnosis across the two treatment conditions. Participants were not excluded from the study if the child met criteria for additional anxiety disorders or for a co-morbid non-anxiety diagnosis. In fact, 84% (*n* = 61) of children met criteria for one or more comorbid anxiety disorder diagnoses and about 43% (*n* = 31) of participants met criteria for a secondary, non-anxiety diagnosis (e.g., ADHD, major depressive disorder, oppositional defiant disorder). The final sample included 73 children and adolescents (Mean age = 8.40 years, 74% male sex^1^) and their parents. Participant, parent, and family characteristics are presented in Table 1.

**Table 1:** Participant characteristics

	M(SD)/*n*(%)		t/χ^2^
	Group (N = 34)	Workshop (N = 39)	
Child age	8.65 (1.63)	8.18 (1.57)	−1.25
Child sex (% male)	26 (76.5)	28 (71.8)	0.21
Number of siblings	1.35 (0.76)	1.43 (1.24)	0.31
Mother education (% completed undergraduate or postgraduate) (n = 28 and 36)	23 (82.1)	21 (58.3)	4.27
Father education (% completed undergraduate or postgraduate) (n = 14 and 16)	12 (85.7)	8 (50.0)	4.95
Mother ethnicity (% white) (n = 26 and 37)	26 (100)	33 (89.2)	3.00
Father ethnicity (% white) (n = 14 and 14)	13 (92.9)	13 (92.9)	2.00
Parental marital status (% parents married) (n = 31 and 37)	28 (90.3)	28 (75.7)	4.51
Parental combined salary (% > $100,000) (n = 30 and 37)	25 (83.3)	29 (78.4)	7.82

All t and χ^2^ p > 0.10

Additionally, as noted below, parents also completed a measure of anxiety symptomology for the participating child’s closest-in-age sibling. Of the 73 participating children, 64 had one or more siblings. Siblings ranged in age from 2 to 26-years-old (M = 8.09, SD = 4.14); 52% were male sex. Of the siblings, 42 (66%) were younger than the target child, 21 (33%) were older, and 1 was a twin. Furthermore, 33 (52%) of the sibling pairs were the same sex and 31 (48%) were the opposite sex. Whether the target child had a sibling or not, the age and sex of the sibling closest in age, and whether the sibling was older or younger or of the same or different sex than the targeted child did not differ significantly between the group and workshop conditions.

**Fig. 1:**
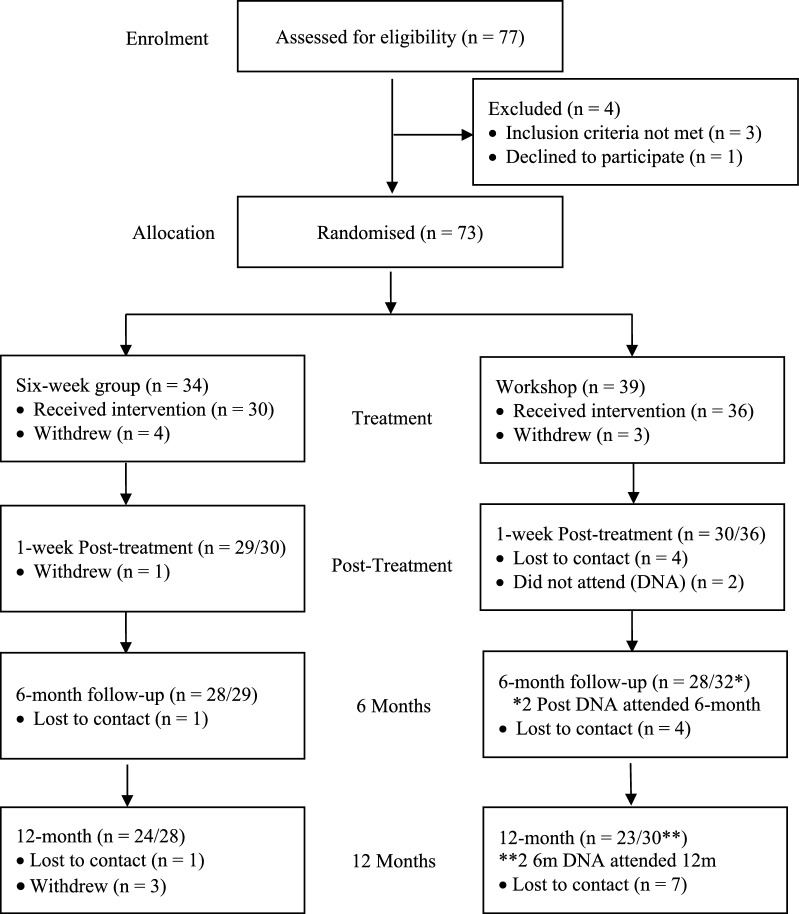
Participant flow. Received intervention: participant received the allocated treatment; withdrew: participant voluntarily withdrew from the study and had no further contact with the study; lost to contact: participant was unable to be contacted by the study; did not attend: participant failed to attend scheduled assessment. Remained in study and attended the next assessment point

Families were given a full description of the study before giving written informed assent/consent. A total of 77 families were tested for eligibility: three families did not meet the inclusion criteria and one family declined to participate. Thus, a total of 73 families were randomized to either the six-week group condition (*n* = 34) or the one-day workshop (*n* = 39). Participant flow through the study is summarized in Fig. 1.

### Procedure

Ethical approval was obtained through the University of Queensland (#2014001727). Families attended an initial (pre) assessment interview at the university’s Psychology Clinic, where informed consent was obtained from all participants. Pre-treatment assessments were conducted by authors VC, IG, MJ and NRA (the first is an experienced clinical psychologist and researcher in the field of child anxiety; the other assessors were postgraduate clinical psychology trainees at the time). Assessments of child anxiety disorder and severity, children and siblings’ anxiety symptoms, parents’ own emotional symptoms, and family-level functioning were completed at four time-points: before treatment, 1-week post-treatment, and at 6- and 12-month follow-up. The assessment battery was completed by the target child’s mother in 79% of the families, by the father in 1% of the families, and by two parents (depending on timepoint) in 20% of the families. Families were compensated with a $20 gift card for each completed follow-up assessment.

Parents were randomly allocated to either the six-week group or one-day workshop condition via a computerized random generator with a 1:1 ratio. Post-treatment and follow-up interviews were completed either face to face or via telephone. Four additional postgraduate clinical psychology trainees served as independent evaluators (IEs) and conducted all follow-up assessments. All IEs were blind to participants’ condition and the design of the study. All interviewers had previous experience in administering the ADIS-IV-C/P, with training having involved a mix of videotaped and live diagnostic interviews. All interviewers met a reliability criterion of 85% in terms of the inter-rater reliability rating for both diagnoses and clinical severity ratings between the trainee and an expert diagnostician (the first author). All interviews were recorded and another trained IE (not involved in conducting any of the interviews) viewed a random twenty percent of interviews over the course of the study in order to ensure there was no interviewer drift. The inter-rater reliability was excellent for the primary diagnosis assigned (K = 0.98).

Of those randomized to treatment, retention of participants at the post-treatment, 6-month and 12-month time-points for the 6-week group was 85%, 82% and 71%. For those assigned to the one-day workshop, the rates were 77%, 72%, and 59%.

### Measures

#### Clinician measures

##### Structured diagnostic interviews with parents

Caseness was determined based on outcomes of The Anxiety Disorders Interview Schedule for DSM-IV for Children—Parent Version [39]. The interview was modified to be consistent with the DSM-5 criteria [38]. Based on parent report, overall child anxiety diagnoses and clinical severity ratings (CSR) were assigned, where a CSR of the primary anxiety diagnosis of 4 or greater on a 9-point scale (moderate to severe) was considered to meet diagnostic criteria. Wherever possible, follow-up diagnostic interviews were conducted face to face at the Psychology Clinic, with a small proportion conducted over the telephone to accommodate parents. Research indicates that administration of the interview via telephone has good inter-rater reliability [40] and good comparability to the face-to face version of the interview [41].

##### Clinical global impressions: improvement scale (CGI-I)

The Clinical Global Impression—Improvement Scale (CGI-I), a seven-point scale ranging from 1 = very much improved to 7 = very much worse [42] was used to determine overall improvements in child anxiety. Scores of 1 and 2 indicate intervention success. Overall mean inter-rater reliability for the team of IEs was excellent (ICC = 0.917).

#### Questionnaire measures

##### Children’s anxiety symptoms

Children completed the Spence Children Anxiety Scale (SCAS [43]); a 45-item self-report measure of anxiety symptomology. It consists of six anxiety subscales that comprise a total score which is reported in the current study. Cronbach’s alpha in this study for the total score was 0.923. Parents also completed the parent version of the Spence Children Anxiety Scale (SCAS-P; [43, 44]) for the child in question (Cronbach's alpha in this study was 0.885).

##### Family-level outcomes

Sibling anxiety, parent anxiety and stress symptoms, and overall family functioning were assessed as secondary outcomes using the SCAS-P, the Depression Anxiety and Stress Scale (DASS-21 [45]), and the Family Assessment Device—General Functioning Subscale (FAD-GF [46]) respectively. Parents were asked to complete the SCAS-P about the sibling closest in age to the ‘identified child’. They also completed the Depression Anxiety and Stress Scale, a 21-item self-report adult measure designed to measure the symptoms of depression, anxiety and stress; the anxiety and stress scores are reported in the current study (Cronbach's alpha in this study was 0.738 [DASS Anxiety], and 0.825 [DASS Stress]). The FAD-GF is a 12-item self-report measure that utilizes a four-point Likert scale (1 = strongly agree and 4 = strongly disagree) to indicate problematic functioning in the family. Lower scores indicate better functioning. Internal consistency in this study was good (α = 0.858).

### Intervention

Triple P—Positive Parenting Program [47] is a public health approach designed to strengthen parenting and support families. It is a multilevel parenting intervention of varying intensities. The intervention is offered at five levels, ranging from a universal public communication campaign on positive parenting (Level 1) to intensive parenting interventions for severe and complex presentations within families (Level 5). Considerable evidence has been found for the efficacy of Triple P [48, 49].

Fear-Less Triple P (FLTP [31, 50]) is a Level 4, Triple P intervention for childhood anxiety. The parent-only CBT intervention consists of a suite of programs allowing for flexibility of delivery. This study investigated the outcomes of delivery modes of the six-weekly group sessions and the one-day workshop format. FLTP is designed to empower parents to take on and enhance their role as the most powerful agent of change for their children. Based on principles of transfer of control and parental modeling, the program teaches parents about effective cognitive-behavioural strategies for managing anxiety, and targets parenting behaviours and family accommodations implicated in the etiology of childhood anxiety (e.g., overprotectiveness, encouragement of avoidance). Thus, it equips parents to ‘coach’ their children in learning cognitive-behavioural strategies for managing anxiety while also focusing on parent–child relationship dynamics in the context of responding to children’s anxiety. Content covered in FLTP includes: psychoeducation about anxiety and parents’ potential role in the maintenance of children’s anxiety; promoting emotional resilience in children; modelling; the role of thoughts in anxiety and mental flexibility; avoidance and exposure; parental strategies for responding to children’s anxiety; and problem solving. Key concepts are incorporated in homework tasks. For example, cognitive restructuring is practiced at home where children are asked by their parents to generate as many interpretations as possible of ambiguous hypothetical child-focused situations.

#### Fear-less triple P group program

The standard FLTP group program consists of six, 90-min weekly sessions (approximately 9 h). Seven groups were run (4–8 families per group) and delivered at the Psychology Clinic (on weekday evenings) by two postgraduate clinical trainee psychologists, trained in the intervention. Supervision was provided by the first author, a licensed clinical psychologist and the lead author of the program. Each weekly session included in-session activities and homework tasks to apply the core concepts and strategies. Families attended an average of 5.4 of the 6 sessions (SD = 0.77). Of the 34 families randomized to the group condition, 29 had only the mother attend each session, two had only the father attend, two had both mother and father attend each session, and one had the mother attend all six sessions while the father also attended two sessions.

#### Fear-less triple P workshop

The FLTP workshop consists of a 1-day program (6 h). It was delivered at the Psychology Clinic by a licensed clinical psychologist and co-facilitated with one or two postgraduate clinical psychology trainees who received training in the intervention. Three workshops were delivered (on weekend days) with each one attended by between 7 and 17 families. Of the 39 families assigned to the workshop condition, 26 had only the mother attend the workshop, three had only fathers attend, and 10 had two parents in attendance.

#### Comparison of the two treatment modes

Both group and workshop formats provided the same core therapeutic concepts, examples and activities; with both incorporating didactic content delivery (via PPT slide presentation and participants’ workbooks). Following treatment, all families were contacted for a brief (15–20 min) phone call one-week post-intervention to give caregivers a chance to review the strategies and problem-solve any concerns arising since program completion. In both the group and workshop modalities, all sessions were recorded. Independent research assistants, who were blind to the study design, reviewed 20% of randomly chosen therapy session recordings for treatment adherence. Of the planned intervention content, 100% was covered as intended in both formats.

The delivery mode of intervention content was the main factor that distinguished the two modes. While both formats used the same PPT slide presentation and the parent workbook as their basis, families in the workshop condition had fewer opportunities to complete activities within the session (with only the most important activities and exercises done in-session) and instead were encouraged to work through these at home. Thus, while families in the workshop condition were engaged in some active learning activities, there was a heavier emphasis on didactic content presentation, with regular check-ins for questions. The conditions also differed in terms of the time allowed for in-group informal discussions. The six-week group program allowed opportunities for parents to not only network but also to discuss knowledge gained during previous weeks and share experiences, whereas the one-day workshop provided relatively little time for this to occur.

### Data analyses

All analyses were conducted in IBM Statistics SPSS Version 25 [37]. Standard significance testing was used to explore all primary and secondary outcomes. Longitudinal, multi-level mixed models were used to explore whether SCAS-P total scores, SCAS-C total scores, FAD scores, sibling SCAS-P,^2^ and parental DASS stress and anxiety scores significantly improved over time and whether there were differences in the changes over time between the treatment conditions. In each model, assessment time points (Level 1) were nested within participants (Level 2) and treatment condition was a Level 2 predictor.

Additionally, Pearson chi-square tests were used to determine whether the number of children who no longer met diagnostic criteria for their primary anxiety disorder or any anxiety disorder differed between the two conditions. Lastly, differences in parent satisfaction ratings at the post-treatment assessment and CGI scores at each of the follow-up assessments were assessed using independent samples *t*-tests.

Missing data at the follow-up assessment time points was accounted for using the multiple imputation procedure in SPSS for all analyses except for the longitudinal, multi-level models. In those analyses, missing data was accounted for by using restricted maximum likelihood estimation which allowed all data at each time point to be utilized without excluding participants who did not complete all measures or attend every time point.

Power analyses indicated that with α = 0.025 and power = 0.80, our acquired sample size was sufficient to detect moderate to large effect sizes, but not small effect sizes.

## Results

See Table 2 for all variable means and standard deviations at each assessment session.

**Table 2:** Variable means and standard deviations across time

	Pre-treatment		Post-treatment		6 month follow-up		12 month follow-up	
	G	W	G	W	G	W	G	W
CSR	5.50 (1.11)	5.56 (1.33)	2.17 (2.56)	2.52 (2.54)	0.48 (1.48)	1.52 (2.55)	0.92 (2.14)	1.15 (2.46)
SCAS-P	34.28 (12.60)	32.70 (14.52)	21.58 (9.83)	27.92 (12.26)	21.39 (7.06)	24.52 (10.78)	21.11 (10.10)	23.03 (14.65)
SCAS-C	28.44 (19.45)	32.18 (16.76)	27.99 (15.02)	30.69 (19.55)	23.33 (14.89)	26.34 (16.51)	22.68 (22.02)	22.63 (19.50)
CGI	–	–	2.14 (0.94)	2.89 (1.03)	1.87 (0.87)	2.09 (1.20)	2.18 (1.25)	1.84 (1.05)
PSS	–	–	62.75 (5.55)	63.01 (6.27)	–	–	–	–
FAD	1.79 (0.48)	1.76 (0.41)	1.81 (0.47)	1.79 (0.48)	1.78 (0.46)	1.77 (0.53)	1.67 (0.51)	1.63 (0.48)
SCAS-P (Sibling)	17.59 (11.96)	19.53 (9.42)	–	–	11.85 (4.13)	13.60 (5.95)	9.93 (4.20)	11.85 (4.61)
DASS-S	6.12 (3.79)	7.29 (4.53)	5.83 (5.76)	6.42 (6.33)	5.84 (7.04)	6.51 (7.80)	5.83 (11.67)	7.35 (13.18)
DASS-A	1.73 (2.65)	2.53 (3.06)	1.10 (2.22)	1.56 (2.56)	0.96 (2.75)	1.76 (3.48)	0.84 (8.40)	0.92 (9.34)

*G* group (N = 34), *W* workshop (N = 39), *CSR* clinical severity rating, *SCAS-P* spence child anxiety scale—parent report, *SCAS-C* spence child anxiety scale—child report, *CGI* clinical global impression—improvement, *PSS* parent satisfaction survey, *CSS* client satisfaction survey, *FAD* family assessment device—general functioning scale, *DASS-S* depression, anxiety, and stress scale—stress subscale;—measure not administered at that session

### Child level outcomes

#### Anxiety diagnoses

**Fig. 2:**
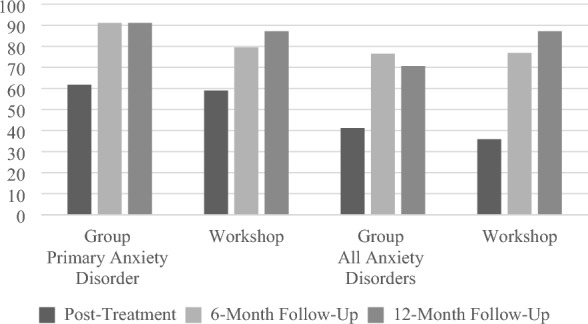
Percentage of participants diagnosis free of their primary anxiety disorder or all anxiety disorders across time

Chi-square analyses indicated that there were no significant differences in the number of children who no longer met criteria for their primary anxiety disorder at post-treatment, χ^2^(1, *N* = 73) = 0.06, *p* = 0.808, 6-month follow-up, χ^2^(1, *N* = 73) = 1.94, *p* = 0.164, or 12-month follow-up, χ^2^(1, *N* = 73) = 0.30, *p* = 0.586, assessments. Of the 39 participants in the workshop condition, 23 (59.0%), 31 (79.5%), and 34 (87.2%) were diagnosis free of their primary anxiety disorder at the post-treatment, 6-month follow-up, and 12-month follow-up assessments, respectively. Of the 34 participants in the group condition, 21 (61.8%), 31 (91.1%), and 31 (91.1%) were free of their primary anxiety disorder at the post-treatment, 6-month follow-up, and 12-month follow-up assessments, respectively (Fig. 2). Mean clinical severity ratings (CSRs) of the primary diagnosis reflect the diagnostic status data and are presented in Table 2.

Furthermore, chi-square analyses indicated that there were no significant differences in the number of children who no longer met criteria for *any* anxiety disorder at the post-treatment, χ^2^ (1, *N* = 73) = 0.21, *p* = 0.644, 6-month follow-up, χ^2^(1, *N* = 73) = 0.00, *p* = 0.964, or 12-month follow-up, χ^2^ (1, *N* = 73) = 3.06, *p* = 0.080, assessments. Of the 39 participants in the workshop condition, 14 (35.9%), 30 (76.9%), and 34 (87.2%) were free of all anxiety disorders at the post-treatment, 6-month follow-up, and 12-month follow-up assessments, respectively. Of the 34 participants in the group condition, 14 (41.2%), 26 (76.5%), and 24 (70.6%) were free of all anxiety disorders at the post-treatment, 6-month follow-up, and 12-month follow-up assessments, respectively (Fig. 2).

#### Anxiety symptoms

**Fig. 3:**
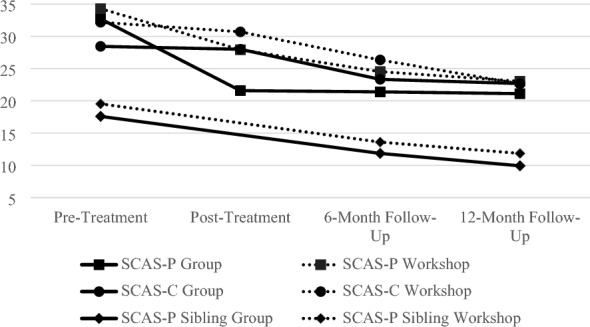
Spence child anxiety scale scores across time

SCAS-P scores significantly reduced over time,* F*(3, 71) = 6.94, *p* < 0.001, and the interaction between assessment session and treatment condition was not significant using the p < 0.025 criterion,* F*(3, 71) = 3.93, *p* = 0.034, indicating that improvement in SCAS-P scores across the four assessment sessions did not differ between the treatment conditions (Fig. 3).

Significance testing indicated that, although on average SCAS-C total scores reduced across time, this reduction was not statistically significant,* F*(3, 71) = 2.33, *p* = 0.088, and did not vary by treatment condition,* F*(3, 71) = 1.04, *p* = 0.386 (Fig. 3).

#### Global improvement

At the post-treatment assessment there was a significant difference in IE rated improvement between the two conditions, *t*(71) = 2.90, *p* = 0.004. Mean CGI scores indicated that participants in the workshop condition were “minimally improved” while participants in the group condition were “much improved.” However, at the 6-month, *t*(71) = 0.75, *p* = 0.455, and 12-month, *t*(71) = -0.99, *p* = 0.323, follow-up assessments the difference between the conditions was nonsignificant and, on average, participants in both conditions were rated as being “much improved.”

### Family level outcomes

#### Sibling anxiety

Mixed-models analysis demonstrated that across the two interventions Sibling SCAS-P scores significantly reduced over time, *F*(3, 71) = 5.49, *p* = 0.009. However, the time by group analysis was not significant,* F*(3, 71) = 1.01, *p* = 0.345, indicating that improvement in SCAS-P scores did not differ between the treatment conditions (Fig. 3).

#### Parental stress and anxiety

Results for parental scores on the stress and anxiety subscales of the DASS were similar; across the two interventions parental stress,* F*(3, 71) = 0.78, *p* = 0.512, and anxiety,* F*(3, 71) = 1.12, *p* = 0.355, did not significantly change over time, nor was there a significant interaction between time and treatment condition for stress, *F*(3, 71) = 0.17, *p* = 0.917, or anxiety, *F*(3, 71) = 0.70, *p* = 0.558. Importantly, parental stress and anxiety scores fell in the “normal” range at the pre-treatment assessment and remained in that range at each of the follow-up sessions.

#### Family functioning

Mixed-models analysis demonstrated that across the two interventions FAD-GF family functioning scores did not significantly change over time,* F*(3, 71) = 2.95, *p* = 0.046, nor was there a significant interaction between time and treatment condition, *F*(3, 71) = 1.02, *p* = 0.395.

### Treatment satisfaction

Parent satisfaction ratings were high at the post-treatment assessment. The difference in parent satisfaction, *t*(71) = 0.183, *p* = 0.855 between the two interventions was nonsignificant.

## Discussion

The present pilot study provides preliminary confirmation for the primary hypothesis that FLTP, a parent-only intervention for childhood anxiety would produce comparable positive diagnostic outcomes when delivered in a one-day workshop format compared to a multi-session group format. Thus, 76.9% and 87.2% of children whose parents were assigned to the workshop were free of any anxiety disorder at 6- and 12-month follow-up respectively, compared to 76.5% and 70.6% of children whose parents were assigned to the 6-week group. Moreover, at post-treatment, 59% and 61.8% of children whose parents were assigned to the one-day workshop and 6-week group conditions respectively were free of their primary diagnosis. These results are consistent with post-treatment results for other parent-focused programs such as the 12-session SPACE program (68.8% diagnosis free [24]) and guided parent-delivered CBT (50% diagnosis free for the full program—two face to face and four telephone sessions—and 39% for the brief program—two face to face and two telephone sessions [21]). Of these two parent-focused interventions, outcomes beyond post-treatment were reported only for the guided parent-delivered CBT intervention (53% free of any anxiety diagnosis for the full guided CBT group and 55% anxiety diagnosis free for the brief guided CBT group at 6-month follow-up [21]). Together, these findings appear to provide additional support for the effectiveness of FLTP [19] both in the 6-week and one-day workshop formats. To the best of our knowledge, the one-day workshop format of FLTP is the first BIC for transdiagnostic anxiety disorders in children to be evaluated. The diagnostic outcomes produced by this protocol represent exciting—if preliminary—new directions.

Data from the questionnaire measures of anxiety symptomatology and the IE-rated improvement scale provide varying levels of support for the effectiveness of FLTP and the lack of difference between the workshop and 6-week group formats. Parental report on the SCAS-P indicated a significant reduction over time, with no difference found between treatment conditions. However, while child reports on the SCAS-C reduced over time, these reductions were not statistically significant. No difference was found between the treatment conditions. It is worth noting that there was a very large range in children’s scores on the SCAS-C (in both treatment conditions and at all time points), as reflected in the large standard deviations. This may have contributed to the failure to find a significant reduction over time.

IE ratings of improvement on the CGI-I indicated that, by the 6- and 12-month follow-up assessments, there was no difference in improvement ratings across the conditions—with all participants, on average, being rated as ‘much improved’. As hypothesized, both formats of FLTP were highly acceptable and satisfactory to families, with no differences found between conditions.

Finally, we explored the impact of FLTP on family-level outcomes. The present study extends the existing literature by showing that both FLTP formats produced comparable positive effects at follow up for the siblings of children with anxiety problems. This finding is important as a significant number of children with diagnosed anxiety problems have siblings with similar problems. If an intervention targeting one child can produce changes in parenting practices that can then be applied to other children in the family, the family level benefits can be considerable. The precise mechanisms through which treatment affected siblings are not clear. Parents may have simply applied the same techniques to different children in the family, siblings may have learned from their siblings’ experiences through observational learning, or reductions in sibling avoidance may have reduced the opportunity for siblings to become anxious in similar situations. Although mean-level family functioning improved from pre-treatment to the 12-month follow-up assessment, there were no significant time or time by condition effects obtained. This was also the case for maternal stress and anxiety scores on the DASS. However, these scores were in the normal range at pre-treatment, with little room for change. Future research could examine the mechanisms through which parenting interventions influence siblings and potentially counter family stress levels when they are observed.

It is important to note that, all findings must be considered in light of the fact that this study was under-powered to detect small between-group differences. Given this limitation, findings in relation to the lack of between-group differences must be interpreted with caution.

Collectively these preliminary findings add to the growing evidence that the systematic targeting of family interactional processes hypothesized to maintain childhood anxiety can be effective treatments in their own right, producing outcomes comparable to “gold standard” CBT interventions for childhood anxiety. The findings are consistent with the wider parenting literature that demonstrates that, for parents of children with conduct problems, low intensity parent-only interventions delivered in a brief, intensive group format or as self-directed online can be as effective as the same content delivered in person in more sessions over a longer time period [49, 51, 52].

Parenting programs that can be delivered in a one-day workshop format are likely to be much more cost effective and accessible for parents as they involve less total time for the parent, lower transportation expenses (less time, fewer trips, reduced parking costs), and reduced likelihood of parents dropping out or missing sessions. However, new funding mechanisms are needed to enable practitioners to be reimbursed for delivering these intensive programs rather than the more customary individual consultation sessions on an hourly basis.

Interestingly, in this study, despite the metropolitan-focused recruitment, over 16% of participant families lived in areas of the state classified as rural and regional—a higher proportion of non-metropolitan families than we have seen in our previous trials. During the assessment process, numerous parents (particularly rural and regional families) anecdotally expressed a preference for the one-day workshop; with many families explicitly requesting assignment to the workshop condition (both before and after assignment) and requiring an explanation of random assignment. Many participant parents reflected on the attraction of a ‘one-off’ intervention, with reduced time, travel and need for childcare the most cited advantages. Relatedly, of two-parent families, a greater percentage had both parents in attendance for the one-day workshop condition compared to the 6-week group condition—reflecting (according to participant feedback) the fact that it is easier to arrange childcare for a single event. Based on these anecdotal observations, it is proposed that a one-day workshop parenting program offers considerable advantages for parents—especially rural and regional parents—compared to a weekly group program (even one as brief as the 6-session FLTP program). The significance of this study lies not in the finding that a 6-h treatment produces similar outcomes to a 9-h treatment; but rather in the finding that a one-day workshop format of FLTP appears to produce similar outcomes to a multi-session group format of the same intervention.

The present findings need to be interpreted in light of the strengths and limitations of our study. Strengths include use of a randomized design, comprehensive outcome assessment including clinical diagnostic measures to establish caseness, multi-informant assessment, a sample with significant non-anxiety comorbidities, high fidelity delivery of both intervention conditions, and inclusion of measures (e.g., family functioning) to study putative mechanism of change. Limitations include the small sample and subsequent lack of power to detect small effect sizes; and recruitment of a sample through community outreach rather than case ascertainment through clinical referral. This latter limitation is mitigated somewhat by the requirement that all participating children met diagnostic criteria and all children experienced significant and interfering levels of anxiety. The study is also constrained by limited data from fathers and the fact that the diagnostic interview was completed by parents only. Participating parents were a relatively homogeneous sample in terms of ethnicity, income, education level and marital status, with an under-representation of more social disadvantaged or minority families. This clearly limits the generalizability of findings. Another important limitation is the attrition rate of participants over follow-up—in particular the workshop participants at the 12-month follow-up point. Data are not available on whether any participating families accessed additional therapeutic services during the follow up period. Although measures of mechanisms of change were included, these were not explored statistically in this paper. This represents an important future direction. Relatedly, it would have been useful to have included a measure of family accommodation in this study—the lack of measurement of this construct is a limitation. It is also noteworthy that our observations about the cost effectiveness of the intervention will require further validation through assessment of the actual costs and benefits incurred by both parents and practitioners. Finally, we do not know whether parent preferences interact with the observed outcomes. It is possible that reception of the preferred delivery format could influence parent satisfaction with treatment or child outcomes.

In a world where childhood anxiety is on the rise; “in person” attendance at health services has become increasingly difficult; and access to mental health services has never been more challenging, two observations in relation to FLTP are worth making. First, an obvious area for future program innovation and evaluation is the development and evaluation of both telehealth delivery and an online format of FLTP. Second, and finally, the advantages and need for an efficacious and brief parent-only program in treating childhood anxiety may never have been more relevant.

## Data Availability

This research meets Level 1 (Disclosure) for all eight aspects of research planning and reporting of the TOP Guidelines as well as Level 2 (Requirement) for data citation, design and analysis transparency, and study and analysis plan preregistration. We report how we determined our sample size, all data exclusions (if any), all manipulations, and all measures in the study, and we follow Journal Article Reporting Standards (JARS) [36]). All data, analysis code, and research materials are available upon request from the authors. Data were analyzed using IBM Statistics SPSS Version 25 [37]. The trial – which was begun in 2015 – was listed with the Australia and New Zealand Clinical Trials Registry (ACTRN 12615001284550), included a basic analysis plan.

## Footnotes

1 Sex was defined in a binary fashion by the biological attributes at birth that are associated with physical and physiological features of female and male sex.

2 Siblings who were under 4-years-old and over 18-years-old (n = 5) were considered outliers and excluded from the analyses presented below. However, the sibling SCAS-P analyses were also run with the outliers included and the results did not differ.
